# Social dominance orientation, intergroup contact and belief in traditional school culture as predictors for parents’ attitudes to school segregation in the Czech Republic

**DOI:** 10.3389/fpsyg.2023.1124781

**Published:** 2023-08-02

**Authors:** Karel Cada, Olga Gheorghiev

**Affiliations:** Department of Managerial Psychology and Sociology, Faculty of Business Administration, Prague University of Economics and Business, Prague, Czechia

**Keywords:** social dominance orientation, school culture, intergroup contact, school segregation, Roma, parents’ attitudes, racism

## Abstract

**Background:**

The over-representation of Roma children in segregated schools is well documented as a prevalent form of institutional racism in the Czech Republic. In the paper, we examine the inclination of parents to support school segregation.

**Objective:**

The paper looks at parents’ preference for school segregation and explores its association to social dominance orientation, intergroup contacts, belief in traditional schooling and the absence of Roma children in school as proof of the school’s good quality. The first hypothesis examines an association between parents’ preference to withdraw their children from ethnically diverse schools and social dominance orientation (one’s degree of preference for inequality among social groups). The second one tests the belief in traditional schooling as a factor contributing to a preference for ethnically motivated withdrawal. The third one studies the extent to which parents’ preference to withdraw their children from ethnically diverse schools is affected by contact with Roma in their everyday life. The final hypothesis tests if parents who view Roma students as an indicator of poor education in a given school are more likely to oppose the presence of Roma students among their children’s peers.

**Methods:**

Quantitative data collection was carried out on a sample of 1,803 respondents. The target group were families with at least one child of primary school age (6–14 years). A binary logistic regression analysis was implemented to assess these relationships.

**Results:**

The study confirmed that ethnically motivated school withdrawal is associated with social dominance orientation, belief in traditional school culture and education. On the other hand, the role of inter-group contact in a school environment was not proved. However, the final statistical model was rather weak explaining approximately 9% of variance in segregation endorsement. The model fit improved significantly when an additional variable – absence of Roma as a sign of a good school – was added. Approximately 15% of the variance in segregation endorsement was explained by the modified set of predictors.

**Conclusion:**

The study argues that ethnically motivated school withdrawal is a result of individual attitudes and situational factors. This means that researchers interested in informal school segregation will need to consider both groups of factors.

## Introduction

Segregation in school education has historically been a topic of particular significance in the Czech Republic. The topic is prevalent in reports on Roma integration (see the Bratinka report from 1997) and was also brought to the attention of the Grand Chamber of the European Court of Human Rights, as well as to a number of other international organisations. Despite growing efforts pushing for the inclusion of Roma students in schools, there is still little visible progress. According to the report of the Czech Ombudsman (2018) and the Ministry of Education, Youth and Sports (hereinafter referred to as the Ministry of Education), there are 77 primary schools in the Czech Republic where Roma students make up more than half of all students in the school. In another 58 schools, they represent between a third and a half of all students.

Although the proportion of children from socially excluded localities being educated in ethnically homogeneous schools is decreasing compared to 2006, when socially excluded localities in the Czech Republic were first mapped (see GAC, 2006), a total of 22% of all students growing up in socially excluded localities are educated in these schools, which are generally perceived as of a lower quality (GAC, 2015). In absolute numbers, this amounts to an estimate of 3,500–5,000 students across the country.

Segregated schooling has been shown to reduce students’ chances of continuing their studies in secondary schools and their chances of entering the open labour market (GAC, 2010). In addition, segregation lowers the performance of the entire education system and places disproportionate demands on teachers’ involvement, leading to work overload (Ombudsman, 2018). The literature on segregation points to its impact on higher crime rates (Weiner et al., 2009), future income (Rivkin, 2000), health (Shen, 2018), and house prices (Clotfelter, 2004). Moreover, the consequences of segregation are borne by students throughout their lives and manifest themselves at all stages of the life cycle (Braddock, 1980; Braddock and McPartland, 1982).

Nekorjak et al. (2011) distinguish three levels of reproduction of school ethnic segregation: (1) spatial segregation, referring to the composition of the municipality, the assignment of schools to certain areas or the total number of schools; (2) the institutional level, defined by strategies opted by schools; and (3) and the individual level, in reference to strategies chosen by individual actors such as parents. This paper focuses on parents’ attitudes to school segregation as a function of several determining parameters.

The study departs from the assumption that parents can reproduce stigmatisation of certain schools both when they choose a particular school for their children and they actively opt out from another. Using the Czech Republic as a case study, Kašparová and Souralová (2014) refer to what sociologist Coleman et al. (1966) called the white flight in reference to white parents withdrawing their children en masse from schools where the proportion of ethnically diverse students increases. The authors show how the increasing homogenization of schools is linked to their stigmatisation and perception by parents as being inferior, slower and of a lower quality.

Lund (2015, 5–6) identifies three key ways through which the selection of particular schools as opposed to others contributes to educational segregation: (1) the selection of schools based on rational choice, (2) the influence of the school’s ethnic composition, and (3) the social anchoring and feelings of the students themselves.

The first pathway is the rational choice of parents to select the school that would best prepare their child for their future educational path. According to the rational choice theory (Breen and Goldthorpe, 1997; Goldthorpe, 1998), parents choose based on cost–benefit evaluations and perceived probability of outcomes among the various educational alternatives available to them. However, options and choice criteria are not universal, and the choices of less privileged students are more constrained than those of more privileged (Ball, 2003; Power et al., 2003; Beach and Dovemark 2011; Reay et al., 2011). As Straková et al. (2017) point out, to place this decision-making in a broader social context, it is important to pay attention to social and cultural reproduction, where more educated parents weigh the benefits and costs differently, estimate the probability of success differently, and define the boundaries within which it is rational to act differently (Glaesser and Cooper, 2014). Thus, higher aspirations and ambitions of parents and children from higher-status families may stem from the way the family thinks about the future and assesses the child’s abilities rather than from the actual costs, returns, and probabilities of success at different levels of education. In a system where the choice of school is administratively restricted, the competence of parents to navigate such a system or the social capital of parents (e.g., the use of fictitious residences of children in the catchment area of the school) conditions the choice of school. Last but not least, parents’ rational choice reflects the rational choice of the school itself. Better-off parents choose better schools (Butler et al., 2013), and when schools can choose the students they admit, they prefer students from better-off families (Burgess et al., 2011).

The second pathway is the ethnic composition of the school. However, this does not imply that parents choose a school based on whether the ethnic composition matches their child’s ethnicity. The ethnic composition of the school serves as a criterion for school quality (Saporito, 2003; Lund, 2015) for all parents, not only those from the majority population (White, 2007; Sikkink and Emerson, 2008). For example, Bifulco and Ladd (2007) show that schools with a percentage of African American students greater than 15% discourage not only majority parents from the ethnic majority, but also African American parents.

The final mechanism of segregation in education described by Lund is the social anchoring and feelings of the students themselves. As Lund shows in his own research on Swedish pupils, children prefer schools with children from the same background. They want to attend school with their friends and they transfer this preference to their parents. School choice is thus not only a matter of practical rationality and pragmatic considerations, but also of social anchoring and feelings (Lund, 2015).

This paper aims at expanding knowledge on factors that influence parents in their decision-making regarding the schools attended by their children. For this purpose, this paper questions the role of proneness to social domination, parents and students’ everyday contact with minority groups, the role of parents’ belief in traditional school and the impact of their perception on the link between the presence of Roma in school and the school’s quality. The effects of parents’ prejudice on school segregation are often studied separately or with intergroup contact as a mediating factor. The paper’s contribution is to link them to parents’ attitudes on school culture and their perception of school reputation. The paper reacts to a missing link on how to connect preferences for inequality and school evaluation schemes in explaining the ethnically motivated school withdrawal (the so-called “white flight”). Our main research question is: how preferences for inequality and preferences for school culture influence parents’ inclination to withdraw their children from ethnically diverse schools? The paper will therefore evaluate four explanatory hypotheses.

The first hypothesis examines an association between the parents’ preference to withdraw their children from ethnically diverse schools and social dominance orientation (SDO), or one’s degree of preference for inequality among social groups. The SDO theory suggests that people with a preference towards social dominance will tend to be also oriented towards ideologies and policies that promote social hierarchies and *vice-versa*, those that are less oriented towards social dominance will favour equality-enhancing policies (Pratto et al., 1994). The theory expects high SDO scoring individuals tend to justify the disadvantage of subordinate groups by endorsing hierarchy-enhancing legitimizing myths (Kteily et al., 2011). The SDO level affects perceived levels of inequality (Kteily et al., 2017) and the high SDO scores are associated with opposition to social welfare, redistributive social policy and civil rights activism (Ho et al., 2012). SDO has been found to positively correlate with prejudicial or discriminatory attitudes towards various social categories (Pratto et al., 1994; Sidanius and Pratto, 1999; Pratto et al., 2006), right-wing authoritarianism (Whitley, 1999; Ekehammar et al., 2004), hostile sexism (Sibley et al., 2007; Roets et al., 2012), SDO appeared as a predictor of attitudes toward people with physical disabilities (Bustillos and Silván-Ferrero, 2013). Despite the plethora of literature on association between SDO and prejudice, few studies have investigated the relation between SDO and specific public policies. SDO was negatively correlated with supportive attitudes toward government-issued international apologies (Mifune et al., 2019). In our case, this would translate into the parents’ tendency to withdraw their children from ethnically diverse schools. There have not been conducted studies researching SDO in the context of Roma in Central and Eastern European countries. We therefore predict that SDO would correlate with a preference for school segregation in relation to Roma children.

The second hypothesis studies the belief in traditional school culture as a factor contributing to the parents’ preference to withdraw their children from ethnically diverse schools. Carrington and Elkins (2002) put the traditional school culture in contrast with inclusion school culture. Traditional school culture frequently emphasizes content rather than students’ needs, facilitates competitions amongst students, does not cater to different learning needs and acknowledges teachers’ strong authority. Hargreaves (2001) describes traditional school culture as fragmented individualism in contrast to collaborative culture. In contrast to individual traditional school culture, some authors put school belongingness as the feeling of connectedness with the school community (Goodenow, 1993; Osterman, 2000; Furrer and Skinner, 2003; Cortina et al., 2017). Traditional school culture is also often mentioned as a barrier to inclusive education (Carrington and Elkins, 2002; Pearce and Forlin, 2005; Lupton and Hayes, 2021). In this context, the traditional school culture is usually studied with respect to teachers’ attitudes, nevertheless, we opted for a separate measurement of belief in the traditional school culture from the point of view of parents. For this purpose, we developed a scale that measures the belief in traditional school culture as expressed in parents’ attitudes to specific school and curriculum characteristics such as focus on competition or the importance of grading.

The third hypothesis tests the extent to which the parents’ preference to withdraw their children from ethnically diverse schools is affected by contact with Roma in their everyday life. The inter-group theory postulates that intergroup contact typically reduces intergroup prejudice, while resentment and conflict tend to develop when groups are isolated from one another. Originally, effectiveness of contact in reducing prejudice has usually been confirmed by research (Hamberger and Hewstone, 1997; Pettigrew, 1997; Wittig and Grant-Thompson, 1998; Gaertner et al., 1999), however, intergroup contact in everyday life rarely occurs under ideal circumstances (see Dixon and Durrheim, 2003). The four most common mediators of the contact effect are in-group norms, out-group norms, intergroup anxiety, and transitive inclusion-of-the-out-group-in-the-self – a process by which one identifies with the other group (Zhou et al., 2019).

The final hypothesis looks at the effect of parents considering the absence of Roma students in schools as an indicator for the school’s quality. Here, we test if parents who view Roma students as an indicator of poor education in a given school are more likely to oppose the presence of Roma students among their children’s peers and indirectly endorse segregation. While this is not an indicator of personal attitudes towards the Roma population, this item expresses segregation endorsement that stems from a concern with quality of education in a specific school.

## Methods

This paper examines the parents’ preference to withdraw their children from ethnically diverse schools and thus segregate Roma students. As predictors of this proneness are considered a high score on the social dominance scale and a strong belief in traditional school culture. The effect of the inter-group contact item and absence of Roma as a sign of good school are also considered.

### The sample

Quantitative data collection was carried out on a sample of 1,803 respondents. The target group were families with at least one child of primary school age (6–14 years). The quantitative data collection was conducted in the form of a quantitative questionnaire survey on the territory of the entire Czech Republic, while observing a quota of predefined features: gender, education, region, and size of the municipality.

The aim of the quantitative data collection was to determine the attitudes of the target group towards education, the evaluation of education within the framework of compulsory primary education and satisfaction with primary schools, as well as to determine the reasons for choosing specific primary schools and opinions on segregation and inclusive education, especially with regard to the education of pupils from socially disadvantaged backgrounds in primary schools. The questions referred to attitudes towards education, evaluation of education within the framework of compulsory primary attendance and satisfaction with primary schools, as well reasons for choosing specific primary schools and opinions on segregation and inclusive education, especially with regard to the education of students from socially disadvantaged backgrounds.

Data collection was carried out by a combined method, where part of the questionnaires was collected through individual standardised face-to-face interviews in the presence of trained interviewers (CAPI), and part of the questionnaires in electronic form were filled in by respondents independently online (CAWI).

Respondents were parents raising at least one child aged between 6 and 14. In relation to education, 33.3% respondents had primary education, 33.3% a high school degree and 33.3% a university degree. The sample was representative for the population of the Czech Republic with respect to regions and size of place of residence. The mean age of the respondents was 40.91 years (SD = 5.83, ranging from 25 to 68 years). Among these respondents, 77% were women.

### Measures

#### The parents’ preference to withdraw their children from ethnically diverse schools (“the white flight”)

As mentioned above, the parents’ preference to withdraw their children from ethnically diverse schools and increased homogenization of schools plays a crucial role in the reproduction of stigmatisation and isolation of schools perceived as being inferior, slower and of a lower quality. This study is interested in the factors that may explain the variation in ethnically motivated school withdrawals. As the “white flight” phenomenon directly implies a segregation of Roma students, the terms “preferences to withdraw their children from ethnically diverse schools” and “preferences for segregation” are used in this study interchangeably.

For this measure, parents were asked about their attitude towards their children having Roma students as their classmates and were given the options “*It is/would be an enriching experience for the students’ collective*,” “*I do not care*” and “*I am against them going to classes with my child*.” The analysis considered the answer “*I am against them going to classes with my child*” and assigned it the value 1, while “*It is/would be an enriching experience for the students’ collective*” and “*I do not care*” were perceived as non-indicators of preferences for segregation and were assigned the value 0.

#### Social dominance orientation (SDO)

For the measurement of social dominance orientation, the 14-item social dominance orientation with the following statements: (1) *It’s OK if some groups have more of a chance in life than others*; (2) *To get ahead in life, it is sometimes necessary to step on other groups*; (3) *If certain groups of people stayed in their place, we would have fewer problems*; (4) *It’s probably a good thing that certain groups are at the top and other groups are at the bottom*; (5) *Inferior groups should stay in their place*; (6) *Sometimes other groups must be kept in their place*; (7) *It would be good if all groups could be equal*; (8) *Group equality should be our ideal*; (9) *All groups should be given an equal chance in life*; (10) *We should do what we can to equalize conditions for different groups*; (11) *We should increase social equality*; (12) *We would have fewer problems if we treated different groups more equally*; (13) *We should strive to make incomes more equal*; (14) *No one group should dominate in society*. The variable was computed as a mean score. The measurement scale was developed by Sidanius and Pratto (1999). In the Czech translation, we have drawn on the work of Loučný (2016), which we have further edited and checked the validity for the Czech sample. The reported Cronbach’s alpha of the social dominance orientation in the Czech sample is 0.86.

#### Belief in traditional school culture

This measure was specifically developed for the purpose of this study. We measured the belief in traditional culture in schools based on respondents’ attitudes towards teaching style, competition, grading and scoring as hallmarks of a good school. Parents were asked about the characteristics of the primary school they would prefer for their children. They were asked to indicate their preference between two opposing characteristics on a scale from 1 to 6, one being the closest value to one characteristic, and 6 to its opposite. A total of nine pairs of opposing school characteristics were included in the question block: (1) *Teachers should be perceived as authorities/ Teachers are mainly to be friends*; (2) *In school, children are to learn in a way that involves self-denial and learning is not just fun/ At school, children are to learn in such a way that they always enjoy learning and feel good about it*; (3) *Scoring and competing with each other motivates pupils to learn/ Scoring and competitions do not belong in school*; (4) *The school should teach mainly according to traditional methods/ The school should introduce modern teaching methods*; (5) *All children should learn the same/ Subject materials should be adapted to the ability of individual students*; (6) *Students should be given daily homework to practice the material at home/ Students should not be given any homework*; (7) *Classes where students are from similar backgrounds work better/ Classes should bring together students from different groups.* The variable was computed as a mean score. The reported Cronbach’s alpha for the items describing the traditional school culture is 0.768. Confirmatory factor analysis showed acceptable fit for this indicator (SRMR = 0.03, RMSEA = 0.07, CFI = 0.94).

#### Contact

Participants’ familiarity with socially vulnerable groups was measured by the presence of Roma students in the classroom attended by the respondents’ children. The parents answered the question “Are there Roma students in the class attended by your child?” The analysis considered the answer “*Yes*” and assigned it the value 1.

#### Absence of Roma as a sign of a good school

We measured the absence of Roma students as a sign of a good school with a single item, whether the respondent chose a school without Roma students as one of the most important characteristics of a good school. This item is not necessarily an indicator of *the parents’ preference to withdraw their children from ethnically diverse schools*, it merely denotes a perceived equivalency between the presence of Roma students and a reported poorer quality of education in that given school. In total, respondents chose from 13 characteristics, including quality of staff, reputation of the school, facilities, standard program and teaching, quality of teaching, access to pupils, and teaching methods. The selected item “a school without Roma students” was assigned with the value 1.

## Results

Prior to the regression model, descriptive statistics and Pearson’s correlations among variables were conducted. Table 1 reported the means, standard deviations, and correlations of the major study variables. *The parents’ preference to withdraw their children from ethnically diverse schools* was positively correlated with social dominance orientation and belief in traditional school culture. Social dominance orientation was positively correlated with the belief in traditional school culture. Contact was positively correlated with *the preference to ethnically motivated withdrawal* and social dominance orientation. No significant correlation was found between contact and belief in traditional school culture. Absence of Roma as a sign of a good school was positively correlated with *the preference to ethnically motivated school withdrawal*, SDO and belief in traditional school culture. However, the correlation values were relatively low.

**Table 1:** Means, standard deviations, and correlations for the main variables.

Variable	The preference to ethnically motivated school withdrawal	SDO	Belief in traditional school culture	Contact	Absence of Roma as a sign of a good school
The preference to ethnically motivated school withdrawal	1				
SDO	0.191**	1			
Belief in traditional school culture	0.117**	0.325**	1		
Contact	0.062**	−0.077**	0.038	1	
Absence of Roma as a sign of a good school	0.252**	0.243**	0.161**	0.065**	1
Mean	0.2464	3.5053	3.418	1.6594	0.1742
S.D.	0.43104	0.8451	0.81822	1.20333	0.37793
Minimum	0	1	1	0	0
Maximum	1	7	6	11	1

*р < 0.05; **р < 0.001.

Table 2 shows that mean scores on the social dominance orientation were decidedly on the low side of the scale, indicating normative disapproval of hegemony. Mean scores on the belief in traditional school culture fell on the high side of the scale, indicating an inclination to support a traditional school culture among participants in the survey. Ethnically motivated withdrawal of their children is endorsed by a minority of respondents – 24.5% of respondents endorsed school segregation. Absence of Roma as a sign of a good school was chosen by the minority (17.4%) of respondents.

**Table 2:** The relationship between the parents’ preference to ethnically motivated school withdrawal, the belief in traditional school culture, SDO and contact.

		The parents’ preference to ethnically motivated school withdrawal (No)	The parents’ preference to ethnically motivated school withdrawal (Yes)	*t*-value	Sig.
SDO	Mean	3.1801	3.6411	−8.268	<0.000
	S.D.	1.01574	1.04547		
	*N*	1,365	447		
Traditional school culture	Mean	3.4458	3.6756	−7.849	<0.000
	S.D.	0.82405	0.88425		
	*N*	1,366	447		
Contact	Mean	1.6162	1.7892	−2.261	0.024
	S.D.	1.08936	1.48868		
	*N*	1,342	446		
Absence of Roma as a sign of a good school	Mean	0.1187	0.3394	−9.220	<0.000
	S.D.	0.32351	0.4740		
	*N*	1,350	453		

Table 2 shows the relationship between *the parents’ preference to withdraw their children from ethnically diverse schools* and their level of belief in traditional school culture, social dominance orientation, contact with Roma in a classroom and the absence of Roma as an indicator of good school. The *t*-test is used to test whether the *preference to ethnically motivated school withdrawal* has a significant difference on social dominance orientation, belief in traditional school culture and contact with Roma in a classroom. Social dominance orientation of people who prefer to *withdraw their children from ethnically diverse schools* is significantly higher than in the rest of the sample. Respondents who tend to withdraw their children are more hierarchic and hegemonic than the rest of the sample. We found a similar effect in relation to belief in traditional school culture. Respondents who tend to withdraw their children form ethnically diverse schools have a stronger belief in traditional principles in education. We also found a significant relation between the preference to separate their children from Roma peers and absence of Roma as an indicator of good school. However, we did not find any significant relation between the preference for segregation and contact with Roma in a classroom.

The association between the parents’ preference to ethnically motivated school withdrawal with occupational groups and education was analysed through chi-square test. No significant association was found in the following groups: senior managers, knowledge workers, qualified professionals (technicians, nurses, etc.), administrative workers, manual workers in services, manual workers and people working in agriculture. Significance association was identified only among unemployed people who inclined significantly towards segregation endorsement (chi-square: 14.247, *p* = <0.001). However, the association is relatively weak – phi coefficient is 0.081 (*p* < 0.001). In relation to education, there was a significant positive association between segregation endorsement and primary education (chi-square: 9.408, *p* = 0.002) and significant negative association with university education (chi-square: 7.682, *p* = 0.006). However, the association is relatively weak – phi coefficient is 0.072 (*p* = 0.002) for primary education and −0.61 (*p* = 0.006) for university degree.

### Binary logistic regression analysis

Table 3 presents the first binary logistic regression model. The Wald test was used to test the set of hypotheses (*H0:* βr = 0vs *H1:* βr ≠ 0) for individual regression slope coefficients. The Wald tests suggested social dominance orientation, primary education were statistically significant at 0.01 and belief in traditional school culture and university degree statistically significant at 0.05.

**Table 3:** Binary logistic regression results (SDO, traditional school culture, contact, education, occupational groups).

Variables	*B*	SD	Wald	df	*p*-value	Odds ratio	95% CI for odds ratio	
							Lower	Upper
SDO	0.373	0.062	36.763	1	0.000	1.452	1.287	1.639
Traditional culture	0.217	0.076	8.050	1	0.005	1.242	1.069	1.442
Contact	0.091	0.045	4.122	1	0.042	1.096	1.003	1.197
Education								
Primary	0.644	0.180	12.865	1	0.000	1.904	1.339	2.707
Secondary	0.178	0.132	1.820	1	0.177	1.195	0.922	1.549
University	−0.328	0.113	8.492	1	0.004	0.720	0.578	0.898
Occupational group								
Senior managers	−0.003	0.394	0.000	1	0.994	0.997	0.460	2.159
Knowledge workers	−0.007	0.393	0.000	1	0.986	0.993	0.460	2.145
Qualified professionals	0.222	0.386	0.332	1	0.565	1.249	0.586	2.660
Administrative workers	0.175	0.384	0.208	1	0.649	1.191	0.562	2.525
Manual services workers	0.188	0.394	0.227	1	0.634	1.206	0.557	2.611
Manual workers	−0.191	0.397	0.231	1	0.631	0.826	0.380	1.799
Working in agriculture	−1.219	0.972	1.573	1	0.210	0.296	0.044	1.985
Unemployed	1.467	0.639	5.279	1	0.022	4.338	1.241	15.169
Constant	−3.546	0.489	52.622	1	0.000	0.029		

In general, people with only primary education were more likely to report the parents’ preference to ethnically motivated school withdrawal than the rest of the sample (*p* < 0.001, OR = 1.904, 95% CI: 1.339–2.707). The parents’ preference to ethnically motivated school withdrawal increased significantly as the preference to social dominance increased (*p* < 0.001, OR = 1.452, 95% CI: 1.069–1.442). The preference to white flight is associated negatively with university education (*p* < 0.05, OR = 0.720, 95% CI: 0.578–0.898). The probability of school withdrawal increased significantly as the belief in traditional school culture increased (*p* = 0.05, OR = 1.242, 95% CI: 1.069–1.442). The model (*χ*^2^ = 110.41, df = 13, *p* < 0.001) was significant. Nagelkerke *R*^2^ was 0.089, and the percentage of correctly classified cases was 75%. Approximately 9% of the variance in segregation endorsement was explained by the set of predictors.

Table 4 presents the second binary logistic regression model. The second model consisted of the same set of variables but the additional variable – absence of Roma pupils as a sign of good school – was added. The Wald tests suggested absence of Roma pupils as a sign of good school, social dominance orientation, primary education and university degree were statistically significant at 0.01 on each variable and traditional school culture and unemployment as occupational group were statistically significant at 0.05 on each variable.

**Table 4:** Binary logistic regression results (absence of Roma as a sign of a good school, SDO, traditional school culture, contact, education, occupational groups).

Variables	*B*	SD	Wald	df	*p*-value	Odds ratio	95% CI for odds ratio	
							Lower	Upper
Absence of Roma as a sign of a good school	1.261	0.138	83.580	1	0.000	3.531	2.694	4.627
SDO	0.284	0.063	20.090	1	0.000	1.329	1.173	1.505
Traditional culture	0.186	0.080	5.473	1	0.019	1.205	1.031	1.408
Contact	0.076	0.046	2.733	1	0.098	1.079	0.986	1.180
Education								
Primary	0.641	0.204	9.884	1	0.002	1.898	1.273	2.829
Secondary	0.208	0.135	2.384	1	0.123	1.232	0.945	1.604
University	−0.328	0.113	8.492	1	0.004	0.720	0.578	0.898
Occupational group								
Senior managers	−0.063	0.397	0.025	1	0.875	0.939	0.431	2.045
Knowledge workers	0.050	0.392	0.016	1	0.900	1.051	0.487	2.267
Qualified professionals	0.257	0.391	0.430	1	0.512	1.293	0.600	2.784
Administrative workers	0.142	0.385	0.136	1	0.712	1.153	0.542	2.453
Manual services workers	0.202	0.403	0.252	1	0.616	1.224	0.556	2.698
Manual workers	−0.221	0.414	0.284	1	0.594	0.802	0.356	1.806
Working in agriculture	−0.991	1.110	0.797	1	0.372	0.371	0.042	3.269
Unemployed	1.685	0.689	5.973	1	0.015	5.391	1.396	20.818
Constant	−3.383	0.495	46.784	1	0.000	0.034		

In general, people considering absence of Roma as a sign of good school were more likely to report a preference to ethnically motivated school withdrawal than the rest of the sample (*p* < 0.001, OR = 3.531, 95% CI: 2.694–4:627). The probability of school withdrawal increased significantly as the tendency to social dominance increased as in the previous model (*p* < 0.001, OR = 1.329, 95% CI: 1.173–1.505). People with only primary education were more likely to withdraw their children from ethnically diverse classes than the rest of the sample (*p* = 0.002, OR = 1.898, 95% CI: 1.273–2.829). This tendency is associated negatively with university education (*p* = 0.004, OR = 0.720, 95% CI: 0.578–0.898). Furthermore, the probability of white flight increased significantly with unemployment (*p* = 0.015, OR = 5.391, 95% CI: 1.396–20.818) and with the belief in traditional school culture (*p* = 0.019, OR = 1.205, 95% CI: 1.031–1.408). The model (*χ*^2^ = 192.939, df = 14, *p* < 0.001) was significant. Nagelkerke *R*^2^ was 0.152, and the percentage of correctly classified cases was 76.7%. Approximately 15% of the variance in segregation endorsement was explained by the set of predictors.

## Discussion

About a quarter of surveyed participants (24.5%) showed a preference to ethnically motivated school withdrawal. At the same time, the findings point to a general disapproval of social hierarchy across respondents, but a rather strong belief in traditional school culture among Czech parents. While the findings indicate a connection between a preference for social hierarchy and belief in traditional school culture and preference to ethnically motivated school withdrawal, this connection is not as strong as one would assume, which suggests the presence of other indirect or hidden factors that may account for the variance in the parents’ strategies.

The study confirmed results of previous studies that school segregation endorsement and ethnic prejudice are associated with social dominance orientation (see Pratto, 1999), belief in traditional school culture (Carrington and Elkins, 2002) and the level of education (Sikkink and Emerson, 2008). On the other hand, the role of inter-group contact (Paluck et al., 2019) in a school environment was not proved. There was no statistical evidence that presence of Roma in the classroom reduced parents’ preference to ethnically motivated school withdrawal. We cannot rule out that the presence of Roma in the classroom increases the opportunities of students to develop inter-ethnic relations and friendships, however, it is not reflected in changes in their parents’ attitudes.

The final statistical model was rather weak, explaining approximately 9% of variance in segregation endorsement. Furthermore, the parents’ preference to ethnically motivated school withdrawal can be hardly explained by parents’ attitudes only. The second model proved the thesis that parents use the proportion of ethnically different children as a criterion for school quality (Saporito, 2003). The absence of Roma as a sign of good school improved model’s fit significantly.

It seems that the parents’ preference to ethnically motivated school withdrawal is derived not only from parents’ attitudes but also from situational and rational behaviour which reflect parents’ everyday heuristics about what is an appropriate school for their children and how to recognize a good school. From the above it follows that parents who tend to withdraw their children from ethnic diverse schools tend to perceive schools with Roma children as of a poor quality in terms education and employment opportunities, non-competitive enough and generally as scoring low in terms of social hierarchy. At the same time, in line with theories of self-fulfilling prophecies, concentrating Roma children and socially vulnerable youth in particular schools necessarily affects the quality of education in these institutions, limits life opportunities of vulnerable youth, and deepens social inequalities.

Practical implications can be derived from the above. In order to reduce the level of school segregation, it will be necessary to communicate with parents. First of all, the reputation of schools which are attended by Roma students should be changed. Parents should have guarantees that these schools provide a good quality education, employ highly qualified staff and have good facilities. However, it seems to be more difficult to overcome barriers on the attitudinal side. These barriers are not based on ethnic prejudices and stereotypes alone, but reflect parents’ deeply held beliefs about how the school system should function and what normative standards it should follow. These beliefs can be problematic not only in relation to Roma students, but can generally affect students and their parents that do not cope well with highly competitive and hierarchical school environments, and prefer instead a more horizontal approach to education. The question then becomes not only about attitudes that parents have towards Roma children, but concerns a more general set of attitudes, strategies and expectations from educational paths.

One of the most important limits of this study is that these results are based on responses from parents only. In reality, however, it is important to note that school choices are rarely strictly in the competence of parents and are rather the result of negotiations with students themselves. What the dataset is missing in this case is a pairing with students’ attitudes, alongside those of parents, which would provide us with a deeper understanding of processes behind educational choices. Another important observation is that prejudice is rarely limited to one sphere only, it overspills to other spheres of real life social interactions. These experiences can be mutually reinforcing in terms of attitudes and expectations. What would make for a more complete dataset in this sense would be questions able to explore manifest or latent racism and discriminatory attitudes in other aspects of life, such as the workplace, personal educational experience, or political values. These new lines of questioning should make for future paths of research.

## Conclusion

Despite a consistently significant level of Roma students’ segregation in schools, the Czech Republic registers insufficient progress towards the mitigation of both structural barriers and individual barriers. In order to advance the knowledge on the nature and extent of various factors of inequality reproduction in education, this paper explores individual levels of segregation reproduction by looking at parents’ attitudes and strategies when choosing an educational path for their children.

This study affirmed that levels of social dominance orientation and belief in traditional school culture have an impact on the parents’ preference to ethnically motivated school withdrawal. It also identified a key role of ethnicity as a sign of school with poor quality. It argues that school segregation endorsement is a result of individual attitudes and situational factors. This means that researchers interested in structural racism will need to consider both groups of factors.

The reproduction of historical and personal experience may play a role. The origins and reproduction of segregation in Czechs schools has been conceptualised both as a remnant of the communist education system (e.g., Amnesty International, 2009), and as a result of local education markets and competition between schools in post-Soviet development. Parents may therefore draw on their own personal experience in school when making a decision for their children.

## Data availability statement

The raw data supporting the conclusions of this article will be made available by the authors, without undue reservation.

## Ethics statement

The studies involving human participants were reviewed and approved by Agentura pro socialni zaclenovani. The patients/participants provided their written informed consent to participate in this study.

## Author contributions

KC led the analytical segment of this paper and the definition of hypotheses. OG looked at literature review and offered support in the analytical part. All authors contributed to the article and approved the submitted version.

## Funding

The work is part of the project Pathways to Work: Educational and professional trajectories of young people from socially excluded environment (TL03000628), which was financed from the state budget by the Technology Agency of the Czech Republic within the ÉTA Program.

## Conflict of interest

The authors declare that the research was conducted in the absence of any commercial or financial relationships that could be construed as a potential conflict of interest.

## Publisher’s note

All claims expressed in this article are solely those of the authors and do not necessarily represent those of their affiliated organizations, or those of the publisher, the editors and the reviewers. Any product that may be evaluated in this article, or claim that may be made by its manufacturer, is not guaranteed or endorsed by the publisher.
